# Nest Modification Protects Immature Stages of the Japanese Orchard Bee (*Osmia cornifrons*) from Invasion of a Cleptoparasitic Mite Pest

**DOI:** 10.3390/insects11010065

**Published:** 2020-01-20

**Authors:** Neelendra K. Joshi, Kusum Naithani, David J. Biddinger

**Affiliations:** 1 Department of Entomology and Plant Pathology, University of Arkansas, 217 Plant Sciences Building, Fayetteville, AR 72701, USA; 2 Department of Biological Sciences, University of Arkansas, Fayetteville, AR 72701, USA; kusum@uark.edu; 3 Fruit Research and Extension Center, Entomology, Pennsylvania State University Biglerville, PA 17307, USA; djb134@psu.edu

**Keywords:** mite, cleptoparasite, Japanese orchard bee, pollinator, pest control

## Abstract

*Osmia cornifrons* (Radoszkowski) (Hymenoptera: Megachilidae) is an effective orchard pollinator. Considering the honey bee population decline in recent years, the conservation and propagation of *O. cornifrons* as an alternative managed pollinator is important in ensuring adequate pollination of tree fruit crops in the eastern United States. A field study was conducted to determine if nest modifications could reduce mite parasites and parasitoid natural enemies that attack managed *O. cornifrons*. Paraffin-coated paper liners (straws) were added to create modified nests, and were compared with the unmodified nests (i.e., nests without paper liners). In each nest, we recorded the number of nest cells with cleptoparasitic mites *Chaetodactylus krombeini* (Baker), and the presence of a parasitoid wasp *Monodontomerus obscurus* (Westwood). We also recorded the number of cocoons, male pupae, female pupae, and unconsumed pollen-nectar provision masses in these nests. Results showed that using paper liners in nest-tunnels greatly reduced the invasion of natural enemies of managed populations of *O. cornifrons*. Most notably, the addition of paper liners provided protection from invasion by *C. krombeini* mites, as the mean number of nest cells with mites were significantly lower in these lined nests compared to the nests without paper liners. A significantly higher number of male and female cocoons of *O. cornifrons* were recorded in the nests with paper liners. The population of *M. obscurus* was almost negligible in this field study. These results suggest that using nests with paper liners may accelerate the *O. cornifrons* population establishment and propagation in commercial orchards of rosaceous fruit crops, and possibly in other crops.

## 1. Introduction

*Osmia cornifrons* (Radoszkowski) (Hymenoptera: Megachilidae), commonly known as the Japanese orchard bee or hornfaced bee, is an important alternative pollinator of various pome and stone fruit crops, such as apples, apricots, cherries, nectarines, peaches, pears, plums, etc. [1,2,3,4,5]. Compared to other managed bees (e.g., *Apis* spp., *Bombus* spp.), populations of *O. cornifrons* are simpler to manage and propagate in commercial agricultural ecosystems [6], but like those bees, it is also adversely affected by several pests and parasitoids [7,8]. In the fruit-growing regions of the eastern United States, *Chaetodactylus krombeini* (Baker) (Acari: Chaetodactylidae), a cleptoparasitic mite, has been the primary pest associated with *O. cornifrons* nests and immature stages [9]. It is a major threat to managed, as well as wild populations of *O. cornifrons* in this region. Its invasion of *O. cornifrons* nests is reported to have adverse impacts on *O. cornifrons* eggs and female larval stages [9], and cause rapid population declines if not managed (DJB, pers. obs.). Two other related mite species attack *Osmia* nests in other regions. *Cybocephalus nipponicus* (Kurosa) has been reported from Japan, where it is known to be a major pest of *O. cornifrons*, managed for apple pollination [1,10,11], and *C. osmiae* (Dufour) is a pest of *O. rufa* in Belgium [12].

*C. krombeini* is also known as a pollen mite (or hairy-footed mite) [13], and primarily feeds on the pollen collected and stored by *O. cornifrons* as a nest cell provision. As a result, the provision in the nest cells is depleted before the completion of larval development, and the mites can also kill eggs and young larvae [14]. In the United States, its native hosts are *Osmia lignaria* (Say) and *Osmia bucephala* (Cresson) [15,16]. Other pests, such as several species of the Torymid wasps of the genus *Monodontomerus*, are prepupal and pupal parasitoids of many megachilid solitary bees, including *O. cornifrons* and the alfalfa leafcutting bee (*Megachile rotundata* (F.)) [17]. The parasitoids consume their hosts and have the potential to reduce *Osmia* populations in the field [18].

Effective management of *C. krombeini* is an essential step toward establishing and maintaining managed populations of *O. cornifrons* for fruit pollination. Different mite management tactics, for instance, applying wintergreen oil and formic acid in the nest of *O. cornifrons* [19], dipping adult *O. cornifrons* in pesticide solutions or treating with lime sulfur [7], using bleach to clean nesting material or substrates [20], and physically removing mites from loose overwintering cocoons by washing them in cold tap water through a sieve (DJB, unpublished data), have been used to control *C. krombeini*. These pesticidal or invasive methods for managing *C. krombeini* may affect nesting behavior or brood success, and may also require technical expertise or restricted pesticides for successful implementation. Manipulating nesting substrates of *O. cornifrons* could be a cost-effective mite management alternative. In this context, the main objective of this study was to examine if modifying nest by adding paper liners to nest-blocks protects the nest provisions and immature stages of *O. cornifrons* from *C. krombeini*, and other arthropod natural enemies generally associated with *Osmia* nests.

## 2. Materials and Methods

### 2.1. Osmia Cornifrons Nests

The effect of nest modification on *O. cornifrons* nesting success was studied in a season-long experiment utilizing a total of 224 potential nest tunnels, including modified (*n* = 112) and unmodified (*n* = 112) nests near an apple orchard in Adams County, Pennsylvania. These nests were housed in four, 56-hole nest-blocks, with 8 mm diameters and 15 cm deep tunnels (Binderboard^®^, Pollinator Paradise, Parma, ID, USA). Binderboard nest-boxes are made of solid wooden boards with drilled holes, which are further cut in half with a lamination on the back. This is so the boards are hinged and open into two halves for easy access to cocoons [21]. We modified the nesting tunnels by inserting paper liners (i.e., straws) in alternate rows in each nest-block so that half the nest-tunnels had paper liners and half did not. The paper liners used were made of paraffin-coated opaque white-colored thin paper (Pollinator Paradise, Parma, ID, USA), and were of the same length (15 cm) as the nest-tunnels in the Binderboard nest-box. These nests were deployed prior to bloom time along the borders of an apple orchard, where wild populations of *O. cornifrons* were nesting in large numbers in old dry apple wood, in a shelter adjacent to a commercial apple orchard and natural forest.

### 2.2. Data Collection and Analysis

After approximately three months from the bloom period, nests were brought to the research station and kept in an outdoor, screened insectary until early winter. Nests were opened and evaluated during mid-winter (late November through December) after the larvae had pupated in the fall, and had developed into overwintering diapaused adults. In each nest-block, at least several tunnels were not provisioned, so only *O. cornifrons* provisioned tunnels were evaluated. In each nest, we recorded the total number of cocoons, male and female cocoons (based on size and location in the nest), unconsumed provision masses (also known as pollen balls), nest cells with mite (*C. krombeini*) establishment, and the presence of the parasitoid *M. obscurus* (Westwood). We also recorded the presence of other nest-associated arthropods. These included the commonly occurring overwintering larvae of *Monobia quadridens* (L.) (Hymenoptera: Vespidae), which is a predator of microlepidoptera, whose larvae overwintered and then pupated in the Binderboards early the following spring. In addition, overwintering pupae of the tree cricket predator, *Isodontia mexicana* (Saussure) (Hymenoptera: Sphecidae), and several species of black ants that overwintered as adults in the nests were recorded. Data in each of the aforementioned categories were analyzed for significant differences using the Kruskal–Wallis rank sum test due to a large number of zeros in the data. Statistical analysis was done in R [22].

## 3. Results

**Figure 1:**
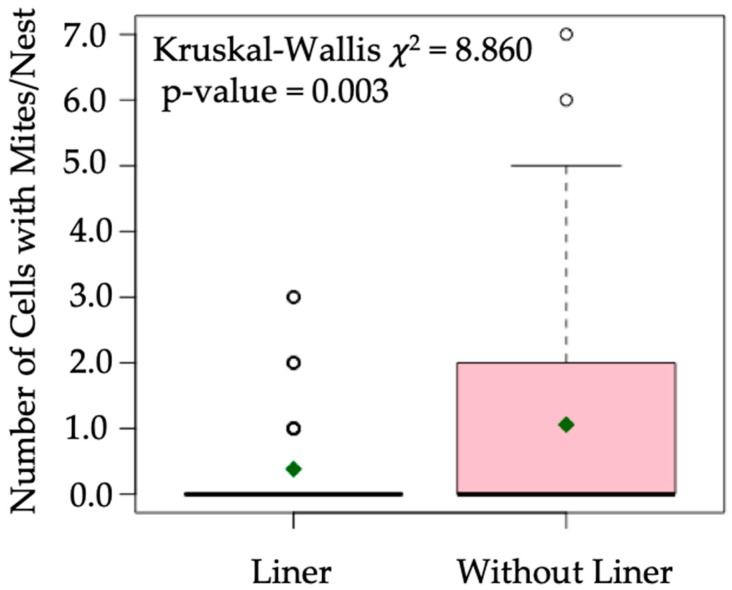
Mean number of mite infested *Osmia cornifrons* nest cells in manipulated nests (i.e., nests with paper liners) and its comparison with the nests without paper liners. The thick horizontal line in the plot represents median, the green diamond indicates mean, and the box indicates the 25th–75th percentile range. Whiskers represent the range of data, while the circles represent outliers. The Kruskal–Wallis rank sum test was performed for significance test (*p*-value < 0.05).

**Figure 2:**
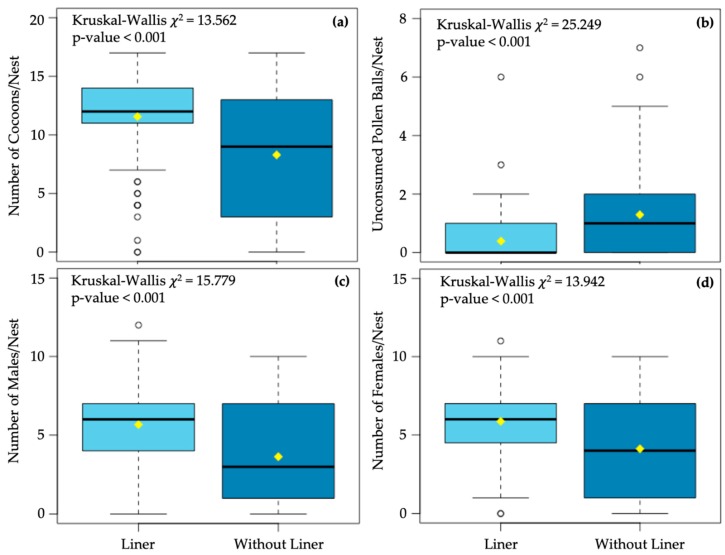
Impact of deploying *Osmia cornifrons* nests with and without paper liners on the mean number of cocoons (**a**), unconsumed pollen balls (**b**), male (**c**), and female (**d**). In each plot, the thick horizontal line represents median, the yellow diamond indicates sample mean, and the box indicates the 25th–75th percentile range. The total range of the data in each plot is shown with whiskers, and open circles represent outliers. The Kruskal–Wallis rank sum test was performed for significance test (*p*-value < 0.05).

Out of 224 potential nest-tunnels in four 56-hole Binderboards, we evaluated 179 individual nests (94 nests with paper liners and 85 nests without paper liners) of *O. cornifrons*. The mean number of *O. cornifrons* nest cells with *C. krombeini* mites were significantly lower (*p*-value = 0.003) in the nests with paper liner (mean = 0.383, se = 0.082) than in the nests without liners (mean = 1.059, se = 0.174) (Figure 1). Mean number of cocoons formed inside each nest with paper liner was significantly (*p*-value < 0.001) higher compared to the nests without paper liner (Figure 2a). Unconsumed pollen balls (e.g., provisions) per nest were significantly lower (*p*-value < 0.001) in the nests with paper liners (Figure 2b). The mean number of males (Figure 2c) and females (Figure 2d) were significantly higher (*p*-value < 0.001) in nests with paper liners than those without liners. The mean numbers of dead cocoons per nest were significantly lower (*p*-value < 0.001) in the nests with liners (mean = 0.021, se = 0.025), compared to the nests without liners (mean = 0.141, se = 0.042). The mean number of *M. obscurus* parasitic wasps per nest was very low (mean = 0.032, se = 0.018 for the nest with liner; and mean = 0.094, se = 0.036 for the nest without liner), but there were no significant differences (*p*-value = 0.141) between the nest types. Among other arthropods associated with *O. cornifrons* nests, *M. quadridens* (mean = 0.129, se = 0.087), ants (mean = 0.024, se = 0.017), and *I. mexicana* (mean = 0.012, se = 0.012) were found only in those nests that were without paper liners.

## 4. Discussion

Associated with *O. cornifrons* and other solitary bee nests, *C. krombeini* mites voraciously feed on the pollen provisioning of the nests, and consequently have detrimental effects on developing larvae of *O. cornifrons* [9] and several other *Osmia* species [18,23]. In this study, it appears that adding paper liners in the nesting tunnels acted as a physical barrier in the nest-to-nest transmission of *C. krombeini,* and consequently protected immature stages of *O. cornifrons* from this mite pest. *C. krombeini* generally disperses from one nest to another nest by hitchhiking on the hairs of adult bees as they emerge from the nests in the spring. However, it could also disperse from nest hole to nest hole by walking through cracks and crevices, particularly via holes on the nest made by other arthropods, such as parasitoid wasps of *O. cornifrons* [8]. Not all larval cells within a single nest-tunnel always have mites, and we speculate that the inclusion of paper liners in modified nests help the provisioning female form a tighter mud seal in the partitions between nest cells, so that movement of mites between nest cells is also restricted.

As evident from this study, selection of appropriate nesting substrates by female bees not only protects them from *C. krombeini*, but may also influence brood success and development. We found that the nest holes with paper liners had advantages for the *O. cornifrons* brood, compared to the nest without paper liners (Figure 2a,c,d). In particular, the nest holes with liners positively influenced *O. cornifrons* life-cycle developmental traits measured in terms of the number of cocoons, and male and female pupae per nest hole. Supplementing nests with paraffin-coated paper liners could be the reason for increased brood success. Nest cell provisioning and nest construction activities (such as plugging the front of the nest, forming a mud partition wall between nest cells, etc.) account for 97% of the total time that a female *O. cornifrons* takes to complete a nest cell [24]. On average, the *O. cornifrons* takes 11.5 trips to collect mud and related materials for constructing mud-wall partitions of a single nest cell [24]. Providing appropriate nesting substrates (such as nests with paper liners) could significantly reduce the amount of time and energy that *O. cornifrons* generally spends on activities, such as sealing the nest and plugging nest cell edges. In lieu of that, *O. cornifrons* would possibly use the saved time and energy (from the nest construction process) in provisioning and egg-laying in the nest cells. Significantly higher numbers of cocoons in nests with paper liners than the nests without paper liners in this study suggest the possibility of such tradeoffs.

Unconsumed pollen balls (i.e., pollen mass provisioning) are found in *O. cornifrons* nests when either eggs are not deposited, the eggs did not hatch, or early instar mortality occurred (DJB pers. obs.). In this study, fewer unconsumed pollen balls per nest indicated higher brood development success in nests with paper liners. Additionally, the mean numbers of dead cocoons per nest were significantly lower in the nests with liners, which is another indication of a higher success in larval development in nests with liners. This lower mortality in nests with paper liners could be due to several interacting factors, such as absence of mites, which ensures an adequate pollen supply to the developing larva.

In this study, fewer incidences of *M. obscurus* parasitic wasps in both types of nest-tunnels (with and without liners) could be due to the use of the Binderboard type of nest-box itself, which is designed to give protection from wasps that are parasitic on *Osmia*. Other arthropods, such as *M. quadridens*, ants, and *I. mexicana,* were found only in those nest-tunnels without paper liners. Though the roles of these arthropods in the *O. cornifrons* nests have not been documented well, their presence in the nests could be a matter of concern. In an earlier study, *M. quadridens* had been accounted for the mortality of developing stages by destroying nest cells, cocoons, and pupae of a related solitary mason bee, *O. lignaria* [25]. Absence of these arthropods in the nests with paper liners is an additional advantage of using paper liners for managing *O. cornifrons* populations. Therefore, adding paper liners to different types of commercially available nesting substrates could be an effective management tactic for aforementioned arthropods. Impact of such management tactics on propagation and conservation of *O. cornifrons* populations in commercial orchards could be a potential area for future research.

## 5. Conclusions

This study demonstrated that modifying the nest of *O. cornifrons* by adding paper liners to nest-tunnels protect *O. cornifrons* from a mite pest (*C. krombeini*) and other arthropod enemies associated with nests. Nest cells with mites were significantly lower in the modified nests compared to the nests without paper liners. Therefore, modification of nesting substrates could be an effective management tactic for various pests of other tunnel-nesting bees, and could be a potential area for future research.

## References

[1] Yamada Y., Oyama N., Sekita N., Shirasaki S., Tsugawa C. (1971). The ecology of the megachilid bee *Osmia cornifrons* and its utilization for apple pollination. Bull. Aomori Apple Exp. Stn..

[2] Xu H.-L., Yang L.-I., Kwon Y.J. (1995). Current status on the utilization of *Osmia* bees as pollinators of fruit trees in China (Hymenoptera: Megachilidae). J. Apic..

[3] Biddinger D., Rajotte E., Joshi N., Ritz A. (2011). Wild bees as alternative pollinators. Fruit Times.

[4] Joshi N.K., Otieno M., Rajotte E.G., Fleischer S.J., Biddinger D.J. (2016). Proximity to woodland and landscape structure drives pollinator visitation in apple orchard ecosystem. Front. Ecol. Evol..

[5] Park M.G., Joshi N.K., Rajotte E.J., Biddinger D.J., Blitzer E.J., Losey J.E., Danforth B.N. (2018). Apple grower pollination practices and perceptions of alternative pollinators in New York and Pennsylvania. Renew. Agric. Food Syst..

[6] Yamada M., Kawashima K., Aizu H. (1984). Population dynamics of the horn faced bee, Osmia cornifrons Radoszkowski, with a special reference to the population management. Bull. Aomori Apple Exp. Stn..

[7] Yamada M. (1990). Control of the chaetodactylus mite, Chaetodactylus nipponicus Kurosa, an important mortality agent of hornfaced osmia bee, Osmia cornifrons Radoszkowski. Bull. Aomori Apple Exp. Stn..

[8] Park Y.L., Kondo V., White J., West T., McConnell B., McCutcheon T. (2009). Nest-to-nest dispersal of Chaetodactylus krombeini (Acari, Chaetodactylidae) associated with Osmia cornifrons (Hym., Megachilidae). J. Appl. Entomol..

[9] McKinney M.I., Park Y.L. (2013). Distribution of Chaetodactylus krombeini (Acari: Chaetodactylidae) within Osmia cornifrons (Hymenoptera: Megachilidae) nests: Implications for population management. Exp. Appl. Acarol..

[10] Qu D., Maeta Y., Goubara M., Nakatsuka K.J., Kozo J., Kenji K. (2002). Reproductive strategy in the two species of cleptoparasitic astigmatid mites, Chaetodactylus nipponicus and Tortonia sp. (Acari: Chaetodactylidae and Suidasiidae), infesting Osmia cornifrons (Hymenoptera: Megachilidae). I. Invasion/infestation patterns and partial use of the host food. Jpn. J. Entomol. New Ser..

[11] Qu D., Maeta Y., Nakatsuka K.J., Kenji K., Goubara M. (2003). Reproductive strategy in the two species of cleptoparasitic astigmatid mites, Chaetodactylus nipponicus and Tortonia sp. (Acari: Chaetodactylidae and Suidasiidae), infesting Osmia cornifrons (Hymenoptera: Megachilidae) II. Life history, phoretic positions, development and reproductivity. Jpn. J. Entomol. New Ser..

[12] Van Asselt L. (2000). Observations on the life cycle of Chaetodactylus osmiae (Dufour, 1839) (Acari: Chaetodactylidae) parasitic on the solitary bee, Osmia rufa (L.), 1758 (Insecta: Hymenoptera) in Belgium. Int. J. Acarol..

[13] Sugden E. (2000). Mitey Bees: The Blue Orchard Bee’s Mite Pest. Scarabogram.

[14] OConnor B., Klimov P. (1962). Chaetodactylus Krombeini Baker. http://insects.ummz.lsa.umich.edu/beemites/Species_Accounts/Chaetodactylus_krombeini.htm.

[15] Baker E.W. (1962). Natural history of Plummers Island, Maryland. XV. Descriptions of the stages of Chaetodactylus krombeini, new species, a mite associated with the bee, Osmia lignaria Say (Acarina: Chaetodactylidae). Proc. Biol. Soc. Wash..

[16] Krombein K.V. (1962). Natural history of Plummers Island, Maryland. XVI. Biological Notes on Chaetodactylus krombeini Baker, a parasitic mite of the megachilid bee, Osmia (Osmia) lignaria Say (Acarina, Chaetodactylidae). Proc. Biol. Soc. Wash..

[17] Eves J.D. (1969). Biology of Monodontomerus Obscurus Westwood, a Parasite of the Alfalfa Leafcutting Bee, Megachile Rotundata (Fabricius) (Hymenoptera: Torymidae; Megachilidae). Master’s Thesis.

[18] Krunic M., Stanisaljevic L., Pinzauti M., Felicioli A. (2005). The accompanying fauna of Osmia cornuta and Osmia rufa and effective measures of protection. Bull. Insectol..

[19] White J.B., Park Y.L., West T.P., Tobin P.C. (2009). Assessment of potential fumigants to control Chaetodactylus krombeini (Acari: Chaetodactylidae) associated with Osmia cornifrons (Hymenoptera: Megachilidae). J. Econ. Entomol..

[20] Bosch J., Kemp W.P. (2002). Developing and establishing bee species as crop pollinators: The example of Osmia spp. (Hymenoptera: Megachilidae) and fruit trees. Bull. Entomol. Res..

[21] Strickler K., Mills J. (2002). Loose Cell Management Systems for Solitary Bees. http://www.pollinatorparadise.com/Binderboards/Osmiabb.htm.

[22] Team R.C. (2013). R: A Language and Environment for Statistical Computing.

[23] Bosch J., Kemp W.P. (2001). How to Manage the Blue Orchard Bee as an Orchard Pollinator.

[24] McKinney M.I., Park Y.L. (2012). Nesting activity and behavior of Osmia cornifrons (Hymenoptera: Megachilidae) elucidated using videography. Psyche J. Entomol..

[25] Byers G.W. (1972). Competitive supersedure by Monobia quadridens in nests of Osmia lignaria. J. Kans. Entomol. Soc..

